# Dynamic modelling and natural characteristic analysis of cycloid ball transmission using lumped stiffness method

**DOI:** 10.1186/s40638-017-0080-4

**Published:** 2017-12-12

**Authors:** Peng Zhang, Bingbing Bao, Meng Wang

**Affiliations:** Department of Mechanical Engineering, Anhui University of Technology, Maanshan, 243000 China

**Keywords:** Lumped stiffness method, Robot joint reducer, Natural frequencies, Vibration modes

## Abstract

The vibration of robot joint reducer is the main factor that causes vibration or motion error of robot system. To improve the dynamic precision of robot system, the cycloid ball transmission used in robot joint is selected as study object in this paper. An efficient dynamic modelling method is presented—lumped stiffness method. Based on lumped stiffness method, a translational–torsional coupling dynamics model of cycloid ball transmission system is established. Mesh stiffness variation excitation, damping of system are all intrinsically considered in the model. The dynamic equation of system is derived by means of relative displacement relationship among different components. Then, the natural frequencies and vibration modes of the derivative system are presented by solving the associated eigenvalue problem. Finally, the influence of the main structural parameters on the natural frequency of the system is analysed. The present research can provide a new idea for dynamic analysis of robot joint reducer and provide a more simplify dynamic modelling method for robot system with joint reducer.

## Introduction

The main inducement of vibration of high-speed robot is robot joint reducer, and therefore, the dynamic research for robot joint reducer is necessary. At present, domestic and overseas scholars have made many deeply research on cycloid ball planetary transmission, including structure principle [1, 2], engagement principle [3], mechanical property [4, 5], and transmission accuracy [6, 7]. However, the dynamic analysis of it has rarely been reported. This paper effectively establishes a simple dynamic model of cycloid ball planetary transmission, which matches with engineering practice. After that, the characteristics of cycloid ball planetary transmission are analysed, and some improvement measures are presented with the purpose of reducing vibration and providing new ideas for robot dynamic analysis.

For the moment, the dynamic models of planetary gear mainly include purely rotational model [8, 9] and translational–torsional coupling model [10, 11]. In purely rotational model, the component’s torsional degree of freedom is only considered. The model is simple because there are few factors are considered. Translational–torsional coupling model also includes the component’s translational degrees of freedom. Compared with purely rotational model, translational–torsional coupling model is more complex, and solving is more difficult. Therefore, it is usually used in theoretical analysis. The result of Ref. [12] shows that when the ratio of support stiffness to mesh stiffness is greater than 10, the simplified purely rotational model and translational–torsional coupling model have some equivalence in the inherent characteristics. For cycloid ball planetary transmission, the translational–torsional coupling model is established, and the inherent characteristic is analysed in Refs. [13, 14]. But the modelling methods are too complex and difficult, especially for a large degree of freedom dynamic system.

In view of that, this paper uses the effectively and simple modelling method—lumped stiffness method to establish the translational–torsional coupling model of cycloid ball planetary transmission. Then, the natural frequencies and vibration modes are revealed by solving dynamic equations of system with the purpose of providing guidance for system design.

## Lumped stiffness modelling

### Structure

**Fig. 1:**
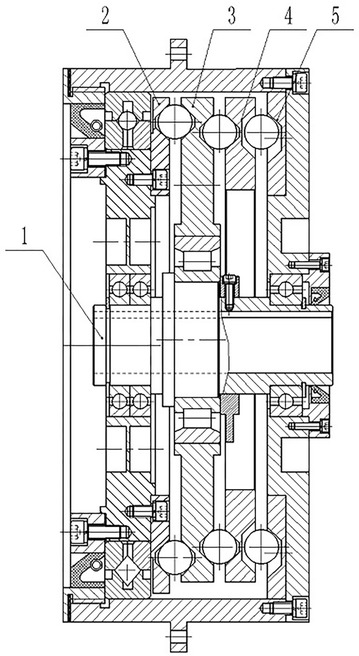
The structure of cycloid ball planetary transmission. 1. Input shaft 2. Centre disc 3. Planetary disc 4. Cross-disc 5. End cover disc

The structure of cycloid ball planetary transmission is shown in Fig. 1. Cycloid ball engagement pairs consist of hypocycloid groove in the left end face of planetary disc, epicycloid groove in the right end face of centre disc, and balls between two discs.

This paper uses cross-ball equal-speed mechanism as output structure for the requirements of robot joint. Cross-ball equal-speed mechanism is made up with the horizontal taper grooves in the right end face of planetary disc, the horizontal taper grooves in the left end face of cross-disc, the taper grooves in the left end face of cross-disc, the taper grooves in the right end face of end cover disc, and balls among three discs. In this paper, cross-ball equal-speed mechanism is proposed, and centre disc is treated as output disc.

### Lumped stiffness model

To simplify the dynamic model, an efficient dynamic modelling method—lumped stiffness method is proposed based on the lumped mass method. The basic thought of lumped stiffness method is as follows: first, the total meshing component force along axis direction will be obtained through mechanical analysis; second, the maximum deformation of meshing point is considered as global deformation, and the component of global deformation along axis direction can be presented; finally, the ratio of total meshing component force to global component deformation along axis direction will be obtained. Obviously, the ratio is lumped stiffness. Compared with the traditional modelling method, the advantages of lumped stiffness method are as follows: nonlinear stiffness, time-varying curvature, and time-varying load have been integrated into the lumped stiffness model and not directly reflected in the dynamic model; the dynamic model will be established and solved easily. The lumped stiffness model of cycloid ball meshing pair and cross-ball meshing pair is, respectively, solved using lumped stiffness method.

**Fig. 2:**
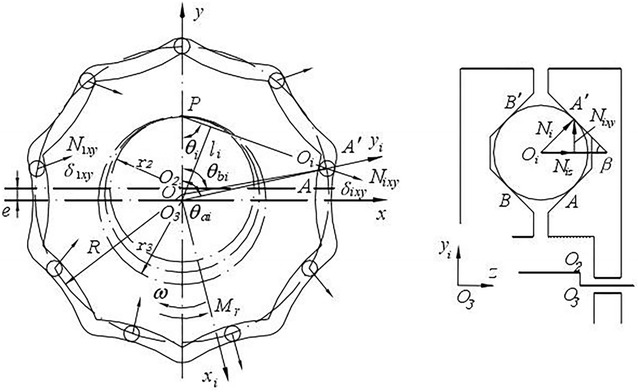
Mechanical model of cycloid ball meshing pairs

The mechanical model of cycloid ball engagement pairs is shown in Fig. 2. Reference [4] shows that the total meshing force of *y* axis is zero, but the total meshing force of *x* axis exists. Therefore, only the lumped stiffness model of *x* axis is needed.

**Fig. 3:**
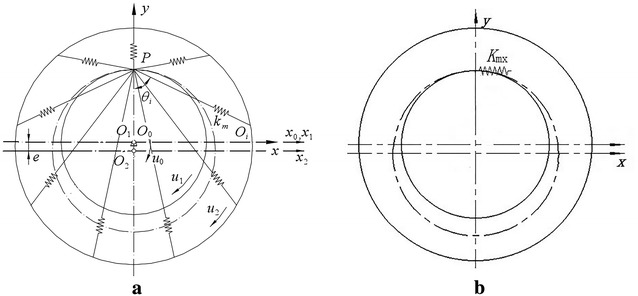
Stiffness model of cycloid ball meshing pairs. **a** Traditional stiffness model. **b** Lumped stiffness model

According to the mechanical model, the stiffness model of cycloid ball meshing pairs can be established as shown in Fig. 3. Figure 3a shows the traditional stiffness model of cycloid ball meshing pairs, (b) shows the lumped stiffness model of cycloid ball meshing pairs. Obviously, the distribution of meshing force is complex. If the meshing forces are not effectively synthesized in modelling, the complexity of modelling will increase. Hence, lumped stiffness model is more convenient and simple compared to traditional stiffness model.

According to the thought of lumped stiffness method, the lumped stiffness of *x* axis is

1 $$\begin{aligned} K_{mx} & = \frac{{\left( {\sum\nolimits_{i = 1}^{{Z_{m} }} {N_{i} \cos \beta } } \right)\left| {_{x} } \right.}}{{(\delta_{1} ,\delta_{2} , \ldots ,\delta_{{Z_{m} }} )_{\rm{max} } \cos \beta \left| {_{x} } \right.}} = \frac{{\left( {\overrightarrow {{k_{m} \delta_{1} }} + \overrightarrow {{k_{m} \delta_{2} }} + \cdots + \overrightarrow {{k_{m} \delta_{{Z_{m} }} }} } \right)\left| {_{x} } \right.}}{{(\delta_{1} ,\delta_{2} , \ldots ,\delta_{{Z_{m} }} )_{\rm{max} } \left| {_{x} } \right.}} \\ & \quad = \frac{{\sum\nolimits_{i = 1}^{{Z_{m} }} {k_{m} \delta_{m} \sin^{2} \theta_{i} } }}{{a\delta_{\rm{m} } }} = \frac{1}{a}k_{m} \sum\limits_{i = 1}^{{Z_{m} }} {\sin^{2} \theta_{i} } \\ \end{aligned}$$

where $\theta_{i}$ represents the angle between the normal line of the *i* meshing point and the *y* axis. $\delta_{m}$ is the maximum deformation in theory, which corresponding to the special location. $\delta_{i\rm{max} }$ is the maximum deformation at any time during the operation. $k{}_{m}$ is the meshing stiffness of single cycloid ball meshing pair. $Z_{m}$ is the number of ball. $\beta$ is the half angle of cycloid groove. $a$ is deformation coefficient, $a = a^{{\prime }} \cdot \overline{{\sin \theta_{i} }}$, $a^{{\prime }} = {{\overline{{\delta_{i\rm{max} } }} } \mathord{\left/ {\vphantom {{\overline{{\delta_{i\rm{max} } }} } {\delta_{m} }}} \right. \kern-0pt} {\delta_{m} }}$, where $\overline{{\sin \theta_{i} }}$ is the average value of the corresponding change interval.

In addition, the torsional angular displacement of discs in cycloid ball meshing pair is generated by meshing displacement. More importantly, the direction of meshing displacement and meshing force are identical. Hence, for the convenience of calculation, the torsional angular displacement is substituted by torsional linear displacement along the direction of meshing force. The lumped torsional stiffness is substituted by lumped stiffness of *x* axis.

**Fig. 4:**
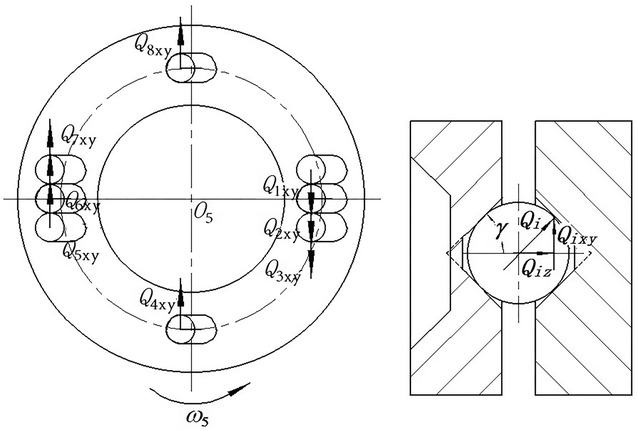
Mechanical model of cross-ball meshing pair

Figure 4 shows the mechanical model of cross-ball meshing pair. The mechanical property of cross-ball meshing pair is analysed in Ref. [15], and its results proposed that the taper grooves along radial direction undertake most of the load, and the taper grooves perpendicular to radial direction hardly undertake the load. In this paper, three taper grooves along the radial direction are arranged on cross-ball equal-speed mechanism with the purpose of improving the bearing capacity. Meanwhile, the taper grooves of side-by-side arranged undertake equivalent load that is $Q_{1xy} = Q_{2xy} = Q_{3xy} ,Q_{5xy} = Q_{6xy} = Q_{7xy}$

**Fig. 5:**
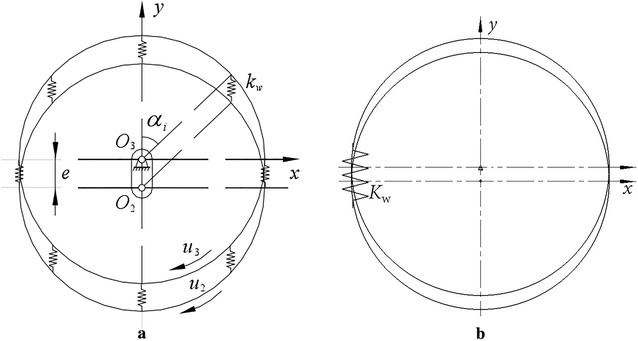
Stiffness model of cross-ball meshing pair. **a** Traditional stiffness model. **b** Lumped stiffness model

The force analysis shows that the total meshing force along *x* axis and *y* axis of cross-ball meshing pair is zero. Hence, there is no need to obtain corresponding lumped stiffness except lumped torsional stiffness. The solution thought is shown as follows: the maximum torsional angular displacement is divided by resultant moment. The stiffness model of cross-ball meshing pair is shown in Fig. 5. (a) shows the traditional stiffness model, and (b) shows the lumped stiffness model. Similarly, the distribution of meshing force is complex. The meshing forces are effectively synthesized in the lumped stiffness model, which is beneficial for dynamic modelling.

Specifically, the lumped torsional stiffness of cross-ball equal-speed mechanism is

2 $$\begin{aligned} K_{w} = \frac{\sum M }{{\Delta \alpha_{i\rm{max} } }} = \frac{{R\sum\limits_{i = 1}^{{Z_{w} }} {Q_{i} \cos \beta } }}{{R\delta_{i\rm{max} } \cos \beta }} \\ = \frac{{3\left[ {Q_{2xy} \left( {R - \frac{e}{2}\cos \phi } \right) + Q_{5xy} \left( {R + \frac{e}{2}\cos \phi } \right)} \right]}}{{\left( {R + \frac{e}{2}\cos \phi } \right)\delta_{6xy} }} \\ = 6k_{w} \\ \end{aligned}$$

where $R$ is the distribution circle radius of taper grooves; $\phi$ is the angle between the straight lines formed by the components and the cross guide rod in the equivalent mechanism of cross-ball equal-speed mechanism; $e$ is eccentric distance of input shaft.

## Translational–torsional coupling model

### Dynamic model

To press close to the physical reality and avoid the complexity of mathematical treatment, the following simplifications and assumptions are made in the dynamic modelling:

- Balls are regarded as elastic element because of the small quality;
- Balls are pure rolling in the grooves, and the influence of friction force is ignored;
- The backlash can be eliminated by clearance screw mechanism, and the influence of backlash nonlinearity is ignored;
- The cross-disc is in a floating state, and the effects of cross-disc are not counted.

**Fig. 6:**
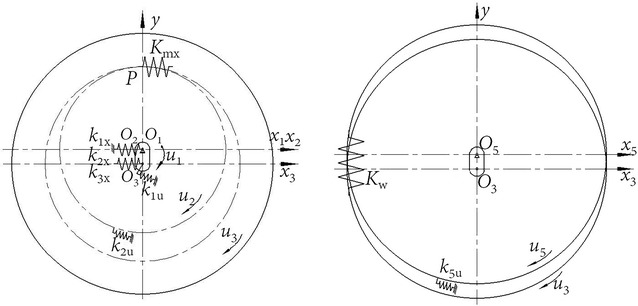
Translational–torsional coupling model of cycloid ball planetary transmission

For the convenience of description of the relationship between the components of cycloid ball planetary transmission, this paper adopts a servo reference system of eccentric shaft (input shaft). Thus, the geometric centre of the input shaft is the coordinate origin. The coordinate system rotates at the speed of input shaft. According to the force analysis, a planar problem is considered where input shaft, centre disc, and planetary disc have two degrees of freedom: one translational around its own axis and one rotational along the *x* axis. End cover disc has one translational degree of freedom. In total, the model has seven degrees of freedom. Figure 6 shows the translational—torsional coupling model of cycloid ball planetary transmission. The sequence number of the components in Fig. 6 is consistent with the sequence number in Fig. 1.

### Relative displacement between components and dynamic equations

The relative displacement between components is clear because the system has fewer components. The specific contents are shown as follows:

1. Relative displacement between centre disc and planetary disc
  3 $$\delta_{23} = x_{2} - x_{3} + u_{2} - u_{3}$$
2. Relative displacement between end cover disc and planetary disc
  4 $$\delta_{53} = u_{5} - u_{3}$$
3. Relative displacement between planetary disc and input shaft
  5 $$\delta_{13} = x_{1} - x_{3} - u_{1}$$

The differential equation of system can be obtained using Newton’s second law:

6 $$\left\{ {\begin{array}{*{20}l} {m_{1} (\ddot{x}_{1} - \omega_{1}^{2} x_{1} ) + k_{3x} \delta_{13} + c_{3x} \dot{\delta }_{13} + k_{1x} x_{1} + c_{1x} \dot{x}_{1} = 0} \hfill \\ {\frac{{J_{1} }}{{e^{2} }}\ddot{u}_{1} - k_{3x} \delta_{13x} - c_{3x} \dot{\delta }_{13x} + k_{1u} u_{1} + c_{1u} \dot{u}_{1} = \frac{{T_{i} }}{e}} \hfill \\ {m_{2} (\ddot{x}_{3} - \omega_{1}^{2} x_{2} ) + K_{mx} \delta_{23} + C_{mx} \dot{\delta }_{23} + k_{2x} x_{2} + c_{2x} \dot{x}_{2} = 0} \hfill \\ {\frac{{J_{2} }}{{r_{2}^{2} }}\ddot{u}_{2} + K_{mx} \delta_{23} + C_{mx} \dot{\delta }_{23} + k_{2u} u_{2} + c_{2u} \dot{u}_{2} = - \frac{{T_{o} }}{{r_{2} }}} \hfill \\ {m_{3} (\ddot{x}_{3} - \omega_{1}^{2} x_{3} ) - K_{mx} \delta_{23} - C_{mx} \dot{\delta }_{23} - k_{3x} \delta_{13x} - c_{3x} \dot{\delta }_{13x} = 0} \hfill \\ {\frac{{J_{3} }}{{r_{3}^{2} }}\ddot{u}_{3} - K_{mx} \delta_{23} - C_{mx} \dot{\delta }_{23} - K_{w} \delta_{53} - C_{w} \dot{\delta }_{53} = 0} \hfill \\ {\frac{{J_{5} }}{{r_{5}^{2} }}\ddot{u}_{5} + K_{w} \delta_{53} + C_{w} \dot{\delta }_{53} + k_{5u} u_{5} + c_{5u} \dot{u}_{5} = 0} \hfill \\ \end{array} } \right.$$

where $J_{\text{i}}$ is the moment of inertia of component $i$ ($i = 1,2,3,5$); $m_{\text{i}}$ is the mass of component $i$ ($i = 1,2,3$); $r_{i}$ is the pitch radius of component $i$ ($i = 1,2,3$), $r_{3} = r_{5}$; $c_{ix}$ is the lateral brace damping coefficient of component $i$ ($i = 1,2,3$); $c_{iu}$ is the torsion brace damping coefficient of component $i$($i = 1,2,5$); $C_{mx}$ is the meshing damping coefficient of cycloid ball meshing pair; $C_{w}$ is the meshing damping coefficient of cross-ball meshing pair; $T_{i}$ is the input torque of input shaft; $T_{\text{o}}$ is the input torque of output disc(centre disc).

The formula (6) is arranged in matrix form:

7 $$\varvec{M\ddot{X}} + (\varvec{C}_{\varvec{b}} + \varvec{C}_{\varvec{m}} )\dot{\varvec{X}} + (\varvec{K}_{\varvec{b}} + \varvec{K}_{\varvec{m}} + \varvec{K}_{\varvec{\omega}} )\varvec{X} = \varvec{F}$$

$$\varvec{X} = [x_{1} ,u_{1} ,x_{2} ,u_{2} ,x_{3} ,u_{3} ,u_{5} ]^{\text{T}}$$

$$\varvec{M} = {\text{diag}}\left( {m_{1} ,J_{1} /e^{2} ,m_{2} ,J_{2} /r_{2}^{2} ,m_{3} ,J_{3} /r_{3}^{2} ,J_{5} /r_{5}^{2} } \right)$$

$$\varvec{F} = \left[ {0,T_{\text{i}} /e,0, - T_{o} /r_{2} ,0,0,0} \right]^{\text{T}}$$

$$\varvec{K}_{\varvec{b}} = {\text{diag}}(k_{1x} ,k_{1u} ,k_{2x} ,k_{2u} ,k_{3x} ,0,k_{5u} )$$

$$\varvec{C}_{\varvec{b}} = {\text{diag}}(c_{1x} ,c_{1u} ,c_{2x} ,c_{2u} ,c_{3x} ,0,c_{5u} )$$

$$\varvec{K}_{\varvec{\omega}} = {\text{diag}}\left( { - m_{1} \omega_{1}^{2} ,0, - m_{2} \omega_{1}^{2} ,0, - m_{3} \omega_{1}^{2} ,0,0} \right)$$

$$\varvec{K}_{\varvec{m}} = \left[ {\begin{array}{*{20}c} {k_{2x} } & { - k_{2x} } & 0 & 0 & { - k_{2x} } & 0 & 0 \\ {} & {k_{2x} } & 0 & 0 & {k_{2x} } & 0 & 0 \\ {} & {} & {K_{mx} } & {K_{mx} } & { - K_{mx} } & { - K_{mx} } & 0 \\ {} & {} & {} & {K_{mx} } & { - K_{mx} } & { - K_{mx} } & 0 \\ {} & {} & {} & {} & {K_{mx} } & {K_{mx} } & 0 \\ {} & {} & {} & {} & {} & {K_{mx} + K_{w} } & { - K_{w} } \\ {\text{sym}} & {} & {} & {} & {} & {} & {K_{w} } \\ \end{array} } \right]$$

$$\varvec{C}_{\varvec{m}} = \left[ {\begin{array}{*{20}c} {c_{2x} } & { - c_{2x} } & 0 & 0 & { - c_{2x} } & 0 & 0 \\ {} & {c_{2x} } & 0 & 0 & {c_{2x} } & 0 & 0 \\ {} & {} & {C_{mx} } & {C_{mx} } & { - C_{mx} } & { - C_{mx} } & 0 \\ {} & {} & {} & {C_{mx} } & { - C_{mx} } & { - C_{mx} } & 0 \\ {} & {} & {} & {} & {C_{mx} } & {C_{mx} } & 0 \\ {} & {} & {} & {} & {} & {C_{mx} + C_{w} } & { - C_{w} } \\ {\text{sym}} & {} & {} & {} & {} & {} & {C_{w} } \\ \end{array} } \right]$$

where $\varvec{X}$ is generalized coordinate array; $\varvec{M}$ is generalized mass matrix; $\varvec{F}$ is external excitation array; $\varvec{K}_{\varvec{b}} ,\varvec{K}_{\varvec{m}} ,\varvec{K}_{\varvec{\omega}}$ are support stiffness matrix, mesh stiffness matrix, and centripetal stiffness matrix; $\varvec{C}_{\varvec{b}}$, $\varvec{C}_{\varvec{m}}$ are support damping matrix and meshing damping matrix. The elements *C*_*mx*_ and $C_{w}$ in the matrix $\varvec{C}_{\varvec{m}}$ have the following form:

8 $$C_{mx} = \frac{1}{a}c_{m} \sum\limits_{i = 1}^{{Z_{m} }} {\sin^{2} \theta_{i} }$$

9 $$C_{w} = 6c_{w}$$

$\varvec{K}_{\varvec{m}}$ is time-varying matrix because the lumped stiffness $K_{mx}$ is a time-varying element with the parameter $\theta_{i}$. To solve the problem conveniently, the $\theta_{i}$ is converted to input shaft angle and the higher-order term is omitted.

10 $$K_{mx} = \frac{1}{a}k_{m} \sum\limits_{i = 1}^{{Z_{m} }} {\sin^{2} \theta_{i} } = \frac{{k_{m} Z_{m} }}{2a} + \frac{{k_{m} Z_{m} }}{2a}\left( {1 - K^{ - 2} } \right)K^{{Z_{m} }} \cos \omega_{1} t$$

where $K$ is short width coefficient of cycloid ball planetary transmission.

The time-varying element of formula (10) is omitted. After the time-invariant $K_{mx}$ is substituted into the mesh stiffness matrix, the dynamic equation of derivative system can be obtained:

11 $$\varvec{M\ddot{X}} + (\varvec{C}_{\varvec{b}} + \varvec{C}^{{\prime }}_{\varvec{m}} )\dot{\varvec{X}} + (\varvec{K}_{\varvec{b}} + \varvec{K}^{{\prime }}_{\varvec{m}} + \varvec{K}_{\varvec{\omega}} )\varvec{X} = \varvec{F}$$

In addition, the mechanical model and stiffness modelling method in this paper are different from Refs. [13, 14], but the mathematical model of cycloid ball planetary transmission is identical.

## Natural characteristic analysis

### Natural frequency and principal mode

The natural characteristic of cycloid ball planetary transmission can be presented by solving the eigenvalue problem of derivative system. The eigenvalue problem of formula (11) is

12 $$(\varvec{K}_{\varvec{b}} + \varvec{K}^{{\prime }}_{\varvec{m}} + \varvec{K}_{\varvec{\omega}} )\varphi_{\varvec{i}} - \omega_{\varvec{i}}^{\varvec{2}} \varvec{M}\varphi_{\varvec{i}} = 0$$

where $\omega_{i}$ is the *i* order natural circular frequency of system; $\phi_{\varvec{i}}$ is the *i* order principal mode of system, $\varphi_{\varvec{i}} = \left[ {\varphi^{(i)}_{{1{\text{x}}}} ,\varphi^{(i)}_{{ 1 {\text{u}}}} ,\varphi^{(i)}_{{ 2 {\text{x}}}} ,\varphi^{(i)}_{{ 2 {\text{u}}}} ,\varphi^{(i)}_{{ 3 {\text{x}}}} ,\varphi^{(i)}_{{ 3 {\text{u}}}} ,\varphi^{(i)}_{{ 5 {\text{u}}}} } \right]$

Without loss of generality, take the cycloid ball planetary transmission used in robot joint as an example, the dynamic characteristics are simulated and analysed. The cross ball equal-speed mechanism is arranged in front of the cycloid ball meshing pair in the prototype. In other words, end the cover disc is fixed and the central disc is used as output component. The speed of input shaft is 1000 *r*/min; the meshing stiffness of single cycloid ball meshing pair is 2.87 × 10^7^ N/m; the meshing stiffness of single cross-ball meshing pair is 4.44 × 10^7^ N/m, and the deformation coefficient *a* is 0.9978. Other basic parameters are shown in Table 1.

**Table 1:** Essential parameters of cycloid ball planetary transmission

Essential parameter	Input shaft	Centre disc	Planetary disc	End cover disc
Number of teeth *Z*		38	40	
Mass/kg	0.854	0.924	2.185	1.447
Moment of inertia *J*_i_/(kg m^2^)	2.69 × 10^−4^	6.69 × 10^−3^	1.44 × 10^−2^	1.19 × 10^−2^
Pitch radius *r*_i_/m	2.5 × 10^−3^	4.75 × 10^−2^	5 × 10^−2^	5 × 10^−2^
Radial stiffness *k*_ix_/(N m^−1^)	5.85 × 10^8^	5.85 × 10^8^	5.85 × 10^8^	
Torsional stiffness *k*_iu_/(N m^−1^)	0	0		1 × 10^9^

By solving the formula (12), the natural frequencies and the principal modes of the system are obtained as shown in Table 2. All natural frequencies are single. The first-order natural frequency is 0, which represents the rigid motion of system. The vibration modes corresponding to the other six-order natural frequencies are both translational vibration and torsional vibration. Furthermore, the approximate results of natural frequencies and principal modes of cycloid ball planetary transmission can be obtained when prototype data in this paper are plugged into the dynamic model of literature [13].

**Table 2:** Natural frequencies and principal modes of cycloid ball planetary transmission

Natural frequency *f*_*i*_/(Hz)		0	708.6	1309.8	2552.8	2705.3	5927.8	6614.9
Principal mode $\phi_{\varvec{i}}$	$\phi^{(i)}_{{ 1 {\text{x}}}}$	0	− 0.0479	0.1962	− 0.2455	− 0.1651	− 0.8094	− 0.6225
	$\phi^{(i)}_{{ 1 {\text{u}}}}$	− 1	− 0.2214	0.2464	− 0.0562	− 0.0312	0.0562	− 0.052
	$\phi^{(i)}_{{ 2 {\text{x}}}}$	0	0.0432	− 0.1024	− 0.3741	− 0.3328	0.6434	0.6365
	$\phi^{(i)}_{{ 2 {\text{u}}}}$	1	− 2.8938	1.85	1.1826	0.8576	0.7569	0.8729
	$\phi^{(i)}_{{ 3 {\text{x}}}}$	1	− 0.4340	2.3444	− 2.7125	− 1.7785	0.0847	− 2.2035
	$\phi^{(i)}_{{ 3 {\text{u}}}}$	0	− 0.2674	− 0.2689	− 0.0445	− 0.1416	− 0.0581	− 0.0665
	$\phi^{(i)}_{{ 5 {\text{u}}}}$	0	− 0.0608	− 0.0759	− 0.2846	0.3459	0.0029	0.0025

### Parametric influence of natural frequency

**Fig. 7:**
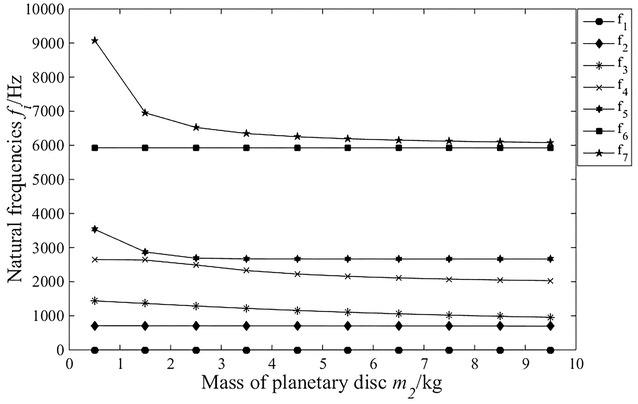
The influence of planetary disc mass on the natural frequencies

**Fig. 8:**
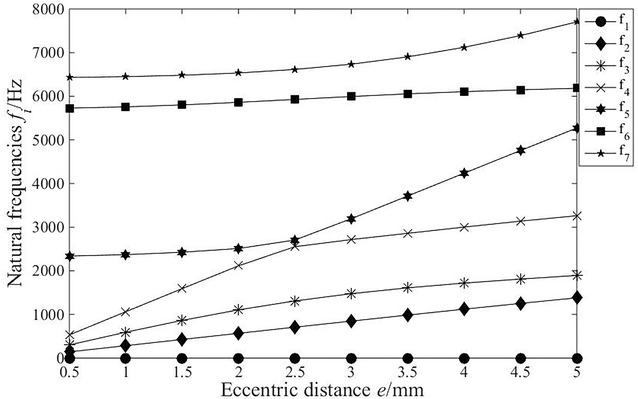
The influence of eccentric distance on the natural frequencies

**Fig. 9:**
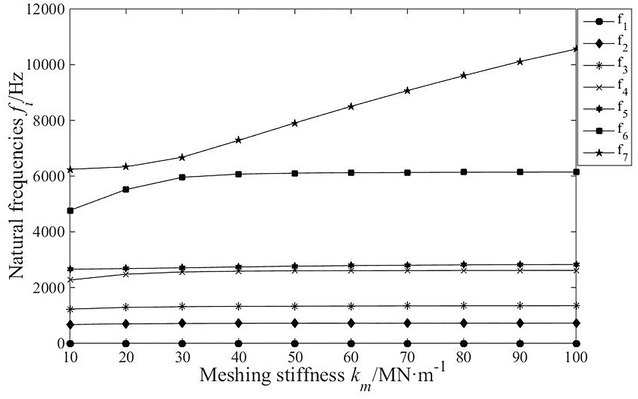
The influence of meshing stiffness on the natural frequencies

**Fig. 10:**
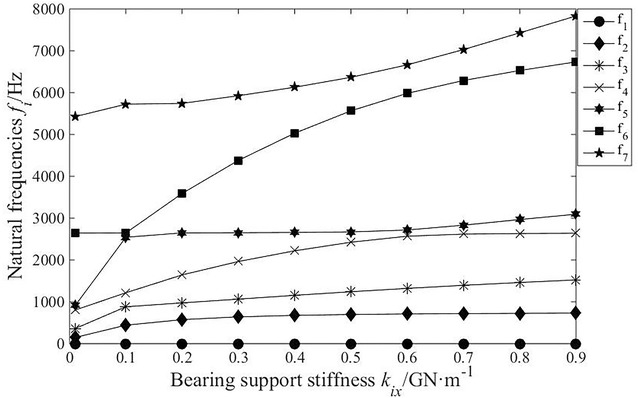
The influence of bearing support stiffness on the natural frequencies

It is necessary to analyse the change regulation of natural frequency relative to parameters of system with the purpose of avoiding vibration. In this paper, based on translational–torsional coupling model, the natural frequency curves of each order are obtained by calculating eigenvalue problem with consideration of main parameters, as shown in Fig. 7, 8, 9 and 10.

As shown in Fig. 7, when the mass of the planetary disc is less than 2.5 kg, the fifth- and seventh-order natural frequencies decrease sharply, and other orders are weakly affected. When the mass of the planetary disc is bigger than 2.5 kg, all the natural frequencies have barely budged.

As shown in Fig. 8, all the natural frequencies increase gradually with the increase in the eccentric, except for the first order. When the eccentricity is 2.5 mm, mode transition appears between the fourth- and fifth-order natural frequencies. At the point of mode transition, the subtle change in parameters will lead to drastic change in natural frequencies. Hence, the sensitive points of parameters should be avoided in the design to avoid drastic change in transmission characteristics.

As shown in Fig. 9, the mesh stiffness has little effect on the first 5 orders natural frequencies of system. The sixth-order and seventh-order natural frequencies increase with the increase in meshing stiffness. When meshing stiffness increases to 3 × 10^7^ N/m, the sixth order natural frequency remains constant, but the seventh order natural frequency rises dramatically.

As shown in Fig. 10, the bearing support stiffness has certain influence on the natural frequencies, except for the first order. When the bearing support stiffness is less than 1 × 10^8^ N/m, the natural frequencies increase obviously with the increase in bearing support stiffness, especially the fifth order; when the bearing support stiffness is greater than 1 × 10^8^ N/m, the sixth- and seventh-order natural frequencies increase significantly. Modal transition phenomenon occurs in the fifth- and sixth-order natural frequencies when bearing support stiffness is 1 × 10^8^ N/m, which should be avoided in the optimization design of system.

## Conclusion

1. To improve the motion accuracy of robot system, the cycloid steel ball planetary transmission used in robot joint is selected as research object. An efficient dynamic modelling method is presented—lumped stiffness method. A translational–torsional coupling model is modelling, and the natural characteristics of system are revealed.
2. All natural frequencies of system are single. The first-order natural frequency is 0, which represents the rigid motion of system. The vibration modes corresponding to the other six-order natural frequencies are both translational vibration and torsional vibration.
3. The number of eccentricity distance and bearing support stiffness may lead to the modal transition phenomenon. The sensitive points of parameters should be avoided as far as possible in the optimization design of system.
